# Attenuation of High Gamma Activity by Repetitive Motor Tasks

**DOI:** 10.1002/hbm.70153

**Published:** 2025-02-07

**Authors:** Takahiro Sanada, Christoph Kapeller, Michael Jordan, Masaharu Miyauchi, Shusei Fukuyama, Teruo Kimura, Satoru Hiroshima, Manabu Kinoshita, Naoki Nakano, Christoph Guger, Naohiro Tsuyuguchi

**Affiliations:** ^1^ Department of Neurosurgery Asahikawa Medical University Asahikawa Japan; ^2^ Department of Neurosurgery Japanese Red Cross Kitami Hospital Kitami Japan; ^3^ G.Tec Medical Engineering GmbH Schiedlberg Austria; ^4^ Department of Neurosurgery Izumi City General Hospital Osaka Japan; ^5^ Department of Neurosurgery Kinki University Sayama Japan; ^6^ Department of Neurosurgery Naniwa Ikuno Hospital Osaka Japan

**Keywords:** attenuation, awake surgery, electrocorticography, epilepsy, functional brain mapping, habituation, high gamma activity

## Abstract

High gamma activity (HGA) is a crucial biomarker for functional brain mapping, particularly in sensorimotor areas, to preserve functionality after brain surgeries. HGA mapping paradigms typically involve multiple task blocks alternating with resting (R) conditions, where each block comprises consecutive tasks under nonresting (NR) conditions. However, the repetitive nature of these tasks may lead to attenuation due to repetition suppression, potentially compromising the accuracy of HGA mapping. This study tests the hypothesis that repetitive grasping paradigms result in attenuated HGA over time in sensorimotor areas. It explores the temporal and spatial characteristics of this attenuation to optimize electrocorticography (ECoG) HGA protocols and enhance result interpretation. Eleven consecutive patients who underwent surgical treatment of intractable epilepsy or malignant glioma were included in this study. Intracranial electrode locations on the pre‐ and postcentral gyrus were considered regions of interest (ROI). Each patient performed ten blocks of ten consecutive grasping trials. The mean z‐scored HGA (60–170 Hz) across these trials was calculated, and attenuation was analyzed using the Kruskal–Wallis test. Obtained signals were also divided into three grouped periods for R and NR groups to assess short‐term attenuation within movement blocks and long‐term attenuation over multiple blocks. Electrode locations were mapped to the MNI152 (Montreal Neurological Institute) brain template to investigate the spatial distribution of attenuation. Distances from each electrode to the hand‐knob region were compared between attenuated and nonattenuated electrodes. A total of 568 electrodes from 11 patients were analyzed, including 139 electrodes within the ROI. Thus, 60 electrodes demonstrated significant HGAs during the grasping task (*p* < 0.05). Sensorimotor HGA z‐scores significantly attenuated over time during both consecutive grasping trials and repeated blocks. Short‐term attenuation (25%, 15/60 electrodes in ROI) was more pronounced than long‐term attenuation (15%, 9/60 electrodes in ROI). Notably, three patients undergoing intraoperative mapping demonstrated less short‐term attenuation compared to long‐term attenuation. Spatially, attenuated electrodes clustered around the hand‐knob region of the precentral gyrus and adjacent areas of the postcentral gyrus. However, no significant differences were observed in the distances from electrodes to the hand‐knob region between attenuated and nonattenuated electrodes. The present study showed that repetitive grasping tasks attenuated the HGA of significant electrodes in the sensorimotor area over time. Considering the findings with the characteristics can further improve the usability of ECoG mapping in terms of more precise results in the most reasonable mapping time.

## Graphical abstract

Repetitive grasping tasks attenuated high gamma activity of significant electrodes in the sensorimotor area over time. Repetition of grasping within each paradigm tended to be more influenced than the number of blocks, and spatial distribution of attenuated high gamma activity showed no significant association with the anatomical hand motor area.

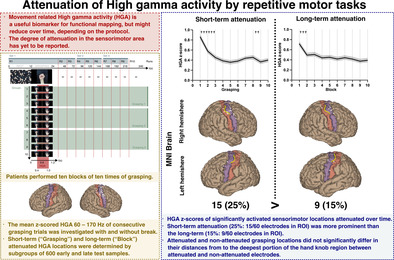

**Summary**

- Repetitive grasping tasks attenuated high gamma activity of significant electrodes in the sensorimotor area over time.
- Repetition of grasping within each paradigm tended to be more influenced than the number of blocks.
- Attenuated and nonattenuated grasping locations did not significantly differ in their distances from to the deepest portion of the hand‐knob region between attenuated and nonattenuated electrodes.

**Abbreviations**
CT: computerized tomography
ECoG: electrocorticography
ECS: electrocortical stimulation
EEG: electroencephalography
FLAIR: fluid‐attenuated inversion recovery
fMRI: functional magnetic resonance imaging
HFB: high‐frequency band
HGA: high gamma activity
MRI: magnetic resonance imaging
PostCG: postcentral gyrus
PreCG: precentral gyrus
SEM: standard error of the mean

## 1 Introduction

Functional brain mapping is a vital procedure for patients with neurosurgical diseases such as epilepsy or brain tumors to preserve their quality of life and, at the same time, to achieve a maximum extent of resection. Electrical cortical stimulation (ECS) is a frequently applied mapping method to localize the human motor cortex (Krause 1909, 1912; Penfield and Boldrey 1937; Penfield and Rasmussen 1950) that evolved to the gold standard over the past decades (Mandonnet, Winkler, and Duffau 2010). Despite its status, its disadvantages due to its technical issues, such as time‐consuming stimulation protocols (Wen et al. 2017), subjective and qualitative interpretation of symptoms (Ritaccio, Brunner, and Schalk 2018), the inability of the patients to express their symptoms, and the risk of stimulation‐induced seizures (Bank, Schevon, and Hamberger 2014). Those issues can be addressed by an alternative technique, utilizing electrophysiological signals, such as electrocorticography (ECoG), a direct marker for neural activity. During movement, subdural recordings from the sensorimotor cortex covered sites with increased power in a high‐frequency band (HFB: 76–100 Hz). This high gamma activity (HGA) co‐localized with movement‐related symptoms during ECS (Crone et al. 1998; Miller et al. 2007). Furthermore, HGA can be associated with the local neuronal firing rate (Ray and Maunsell 2011) and, thus, interpreted as a generalized marker for brain functions, including language (Crone, Hao, et al. 2001; Wu et al. 2011), auditory perception (Crone, Boatman, et al. 2001; Edwards et al. 2005), and visual processing of faces (Sanada et al. 2021; Schalk et al. 2017). Recent studies showed that HGA mapping can overcome several issues of ECS being recognized as a valuable technique characterized by reduced mapping time, objective and quantitative results, and less risk of induced seizures (Kapeller et al. 2015; Ogawa et al. 2014, 2017; Tan et al. 2024). However, HGA mapping is still an emerging technology and not yet a complete alternative to ECS mapping (Arya et al. 2015, 2018; Kapeller et al. 2015; Ogawa et al. 2014, 2017; Tamura et al. 2016).

HGA mapping does not require electrical stimulation but relies on tasks defining brain maps' contrast. Mapping protocols are usually designed in blocks of one or more tasks, which must be repeated to accumulate enough data for statistical analysis. However, a paradigm design with repeated tasks can cause repetition suppression in HGA during repeated working memory (Merzagora et al. 2014), face recognition (Rangarajan et al. 2020), and visual tasks (Matsuzaki et al. 2012) in ECoG. This simple way of nonassociative learning likely causes decreased HGA (Christoffersen 1997; Harris 1943). Meanwhile, the fundamental neural process of habituation is a neural adaptation, which is a decreased neural activity by repetitive stimuli (Verhoef et al. 2008). This effect varies across brain regions (Matsuzaki et al. 2012; Weiner et al. 2010), raising the question of whether the attenuation of HGA in sensorimotor areas‐crucial functional regions for postoperative preservation affects the interpretability of mapping results. Furthermore, HGA mapping paradigms typically require multiple task blocks interspersed with resting (R) condition, and each block encompasses the consecutive tasks with nonresting (NR) condition. Understanding how HGA evolves over time under these conditions could optimize task design, minimize mapping duration, and enhance interpretability by identifying spatial patterns of HGA attenuation.

Hence, the current study attempted to test the hypothesis that repetitive grasping paradigms cause attenuation of the HGA over time in sensorimotor areas. We further investigated the temporal and spatial characteristics under R and NR conditions to seek an optimal ECoG HGA protocol and further interpretation of the results.

## 2 Methods

### 2.1 Patients

Eleven consecutive patients at the Asahikawa Medical University Hospital and Kindai University Hospital underwent surgical treatment of intractable epilepsy or malignant glioma between 2019 and 2022. Three male and eight female patients (*n* = 11), whose ages ranged from 17 to 65 years, underwent either chronic implantation of subdural electrodes for seizure monitoring or temporary implantation during an awake surgery, guiding curative surgical treatment. The patients' characteristics are listed in Table 1. The Institutional Review Board of the Asahikawa Medical University (Asahikawa Medical University Research Ethics Committee) and the collaborative institute (Kinki University Research Ethics Committee) approved the study. Written informed consent, including a detailed explanation, was obtained from each patient and their family. Each patient's brain was reconstructed in FreeSurfer (Martinos Center for Biomedical Imaging, Cambridge, MA, United States) using the T1‐weighted MRI, except for Patient 5 for whom Fluid‐attenuated inversion recovery (FLAIR) images before the implantation were used due to the lack of T1‐weighted images (Dale, Fischl, and Sereno 1999).

**TABLE 1:** Demographic data for all 11 patients.

Patient	Institution	Age	Sex	Surgery	Electrode implantation	Handedness	Covered hemisphere	Total electrodes (*n*)	ROI electrodes (*n*)	Tested electrodes (*n*)	Trials (*n*)
1	AMUH	65	Male	Awake	Temporaly	R	Left	60	8	8	1
2	KUH	45	Female	Awake	Temporaly	R	Left	64	20	19	1
3	KUH	45	Female	Epilepsy	Chronic	R	Right	40	16	16	1
4	KUH	45	Male	Awake	Temporaly	L	Right	20	10	10	1
5	KUH	48	Female	Epilepsy	Chronic	R	Right	58	14	14	3
6	KUH	26	Female	Epilepsy	Chronic	R	Right	12	8	8	2
7	AMUH	36	Female	Epilepsy	Chronic	R	Left	44	11	11	3
8	KUH	23	Male	Epilepsy	Chronic	R	Right	60	18	18	1
9	KUH	55	Female	Epilepsy	Chronic	R	Right	44	8	8	1
10	KUH	17	Female	Epilepsy	Chronic	R	Both	62	12	11	1
11	KUH	22	Female	Epilepsy	Chronic	R	Right	104	16	16	1

Abbreviations: AMUH, Asahikawa Medical University Hospital; KUH, Kinki University Hospital.

Intracranial EEG was obtained from subdural grid electrodes (Unique Medical Co. Ltd., Japan) with 1.5–3.0 mm exposed diameter and 5–10 mm spacing.

### 2.2 Temporary Subdural Implantation (Awake Surgery)

Three patients underwent implantation of subdural grid electrodes on the primary motor and sensory cortex for intraoperative mapping during awake surgery. ECoG recording was performed during an awake craniotomy before the lesion resection. Photographs of the electrode grids on the exposed brain were taken to obtain the sensor locations and channel assignment for the intraoperative mapping. The locations of the electrodes were defined via photograph‐MRI‐co‐registration (Dalal et al. 2008; Gupta et al. 2014).

The first author, a board‐certified neurosurgeon with 8 years of clinical experience, drew the anatomical structures on the intraoperative photographs, which were used as an electrode montage during intraoperative mapping. The skeletonized photographs with anatomical lines and electrodes on the brain surface were overlaid over the 3D brain with the specified surgical angle. Then, the electrodes were projected on the 3D brain based on the co‐registered information. The procedure is shown on the top workflow (Figure S1) and performed by the in‐house software cortiQ Montage Creator (g.tec Medical Engineering GmbH, Austria).

### 2.3 Chronic Subdural Implantation (Epilepsy Monitoring)

The presurgical assessment of eight patients with epilepsy required the implantation of subdural grid electrodes on the primary motor and primary sensory cortex for diagnostic purposes, including video electroencephalography (EEG) monitoring to localize seizure onset zones and routine functional mapping. Each patient had a preoperative computerized tomography (CT) scan to identify electrode locations in conjunction with preoperative magnetic resonance imaging (MRI) for part of diagnostic purposes.

The postoperative CT scans were co‐registered with the MRI scans using the cortiQ Montage Creator software (Mattes et al. 2001, 2003; Thevenaz and Unser 2000). Then, electrode artifacts in the CT were thresholded and projected to the cortex surface as shown in the bottom workflow (Figure S1).

### 2.4 Data Acquisition and Paradigm Designs

During awake surgeries, the patients' bispectral index (BIS) level was kept beyond 90 to be awake during the mapping session. ECoG was obtained at the beginning of the awake phase and before the resection. For epilepsy cases, the HGA mapping was conducted at the bedside once the epilepsy monitoring was completed. Electrode locations on the pre (PreCG) and postcentral gyrus (PostCG) were considered to be ROI.

A 14‐in. presentation monitor was placed at 80 cm in front of each patient's face, showing visual stimuli to the patients, who were instructed to look at the screen and focus on the paradigm while ECoG was recorded. Data were obtained by a DC coupled g.HIamp biosignal amplifier (g.tec Medical Engineering GmbH, Austria) and digitized with 24‐bit resolution at a sampling rate of at least 1200 Hz. Subdural electrodes outside the ROI served as ground (GND).

**FIGURE 1:**
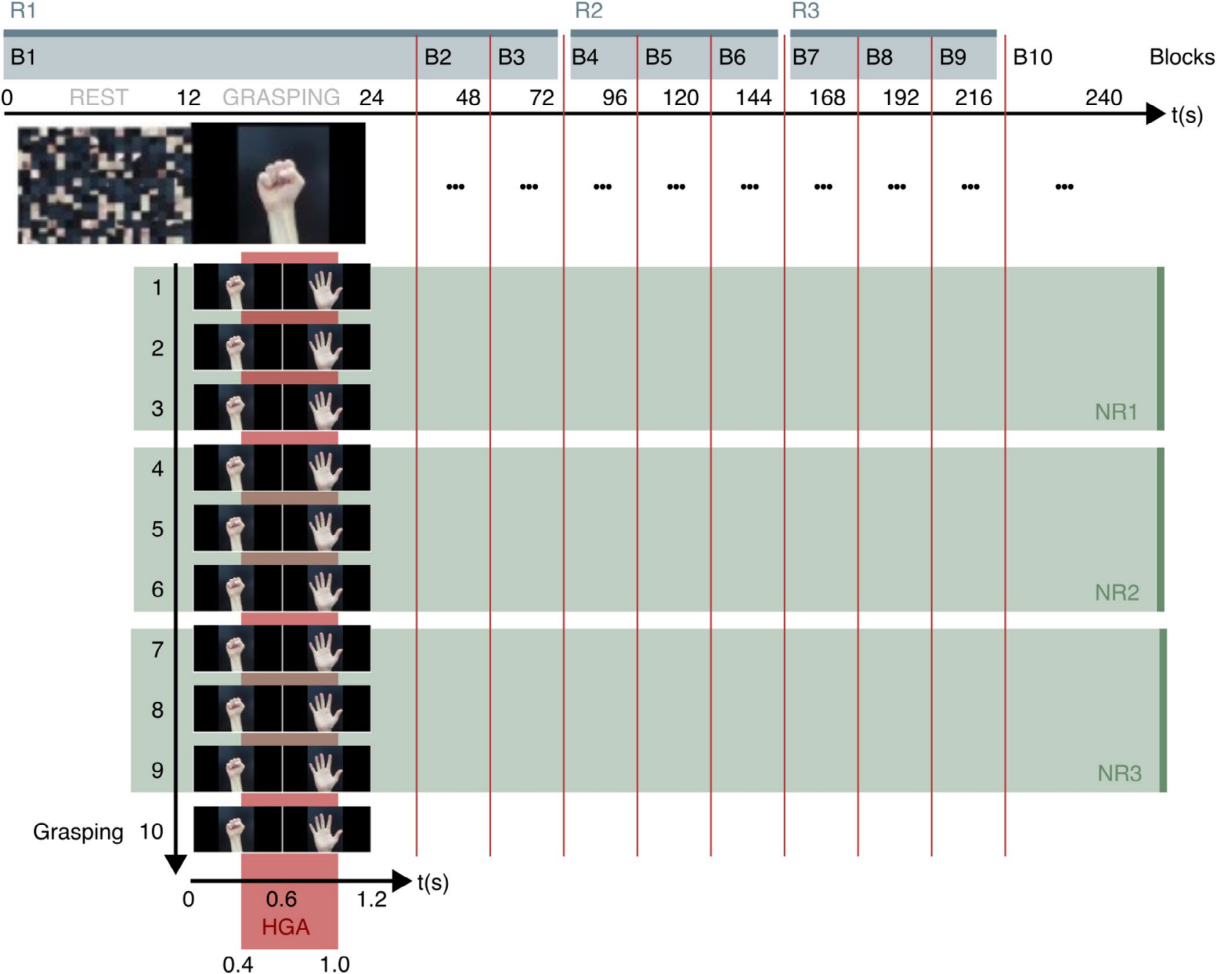
Paradigm design. Experimental design with 10 blocks (B1‐B10), each consisting of a 12‐s rest block, followed by 10 grasping trials. Grasping trials were divided into 3 groups, Nonresting (NR)1–3. Blocks were divided into 3 groups, resting (R)1–3.

Each patient was first asked to stay relaxed for a 10‐s preduration. Afterward, ten blocks (B1 to B10) were performed, each consisting of a 12‐s rest phase (baseline) and a consecutive 12‐s grasping (Figure 1). During the grasping task, the patients were instructed to “close the hand” for 0.6 s (600 ms) followed by “open the hand” for 0.6 s (600 ms). All blocks together took a total of 4 min and 10 s. Patients were asked to open and close the contralateral hand depending on the covered hemisphere. The bilateral case P10 had coverage of the left motor cortex and was asked to move the right hand.

Obtained signals were also divided into three groups over time for nonrest (NR) and rest (R) groups for each electrode and patient and analyzed to investigate the degree of short‐term and long‐term HGA attenuation (Figure 1). Here, short‐term means for the period of repeated movements without a break, and long‐term means over a period including breaks. Specifically, the NR 1–3 were selected for testing the HGA intensity in early trials against later trials after consecutive movements within a block, whereas the R 1–3 were used to reveal whether HGA intensity recovers during breaks of 12 s or if any changes remain permanent throughout the experiment. The 10th trial in each grasping and the 10th block were excluded to better balance the data in the subgroups.

### 2.5 Signal Processing and Statistical Evaluations of HGA

Initial processing steps included a remove drift filter (2 Hz high‐pass, fourth‐order Butterworth) and a common average reference. Next, a band‐pass filter (60–170 Hz, Butterworth, low and high pass of the fourth order each) was applied, followed by a Hilbert transform to obtain broadband gamma signals, which were downsampled (to 400 Hz). Bandpower values were obtained in 100 ms epochs, log‐transformed to approximate a Gaussian distribution of band power samples, and further standardized to z‐scores based on the mean and standard deviation of band power samples during the 12‐s resting periods. HGA, broadband power in high gamma frequencies 60–170 Hz, was calculated from intracranial electrodes and measured from 0.4 to 1.0 s (400–1000 ms) window following task presentation (Figure 1). For each electrode, a *t*‐value was obtained testing whether resting trials and hand movement trials have the same mean HGA. Thus, *p*‐values were taken from a t‐table and Bonferroni‐corrected for the number of electrodes in each case. Electrodes within the ROI, showing significant HGA (*p* < 0.05) for grasping, were further selected for the attenuation test.

### 2.6 Temporal Dynamics of HGA

The mean HGA z‐score of the first grasping trial across blocks was tested against every other average trial as a first attenuation test. The same test was performed backward, where the last block was tested against every other. A Kruskal–Wallis test with Dunn's multiple comparison test was performed on the HGA of each electrode to identify the significant difference in the intensities (*p* < 0.05).

Finally, temporal dynamics of HGA z‐scores were tested for early against late trials, specifically by calculating the mean HGA z‐score and two standard errors of the mean (SEM) of the first and ninth grasping trials of each block and to observe the slow attenuation over blocks, of the whole first block B1 against the whole last block B10. Additionally, the dynamics were obtained for the grouped data as well, reducing the standard error by maximizing the test data.

### 2.7 Spatial Distribution of Attenuated Electrodes and MNI Brain Creation

To delineate the spatial characteristics of the electrodes influenced by attenuation, a Kruskal–Wallis test with Dunn's multiple comparison test was performed on the HGA of each electrode to identify the significant difference within the NR and R groups, respectively. Additionally, a channel was defined as attenuated if NR group 3 was significantly lower than NR group 1. A *p* < 0.05 was considered significant.

We subsequently investigated the spatial distribution of the attenuated sites by projecting the electrode locations of all patients onto the MNI152 (Montreal Neurological Institute) brain using the spherical registration output from FreeSurfer (Fischl et al. 1999). The hand‐knob region was identified as an inverted omega shape, directed posterolaterally, and protruding into the central sulcus in the MNI brains (Yousry et al. 1997).

The relationship between the total number of the PreCG and the PostCG in both NR and R groups was investigated by Fisher's exact test. Finally, we calculated the distance from each electrode to the deepest portion of the hand‐knob region (sigma) and compared the distances between attenuated and nonattenuated electrodes in both NR and R groups using the Mann–Whitney *U* test. A *p* < 0.05 was considered significant.

## 3 Results

### 3.1 Electrodes in the ROI

**FIGURE 2:**
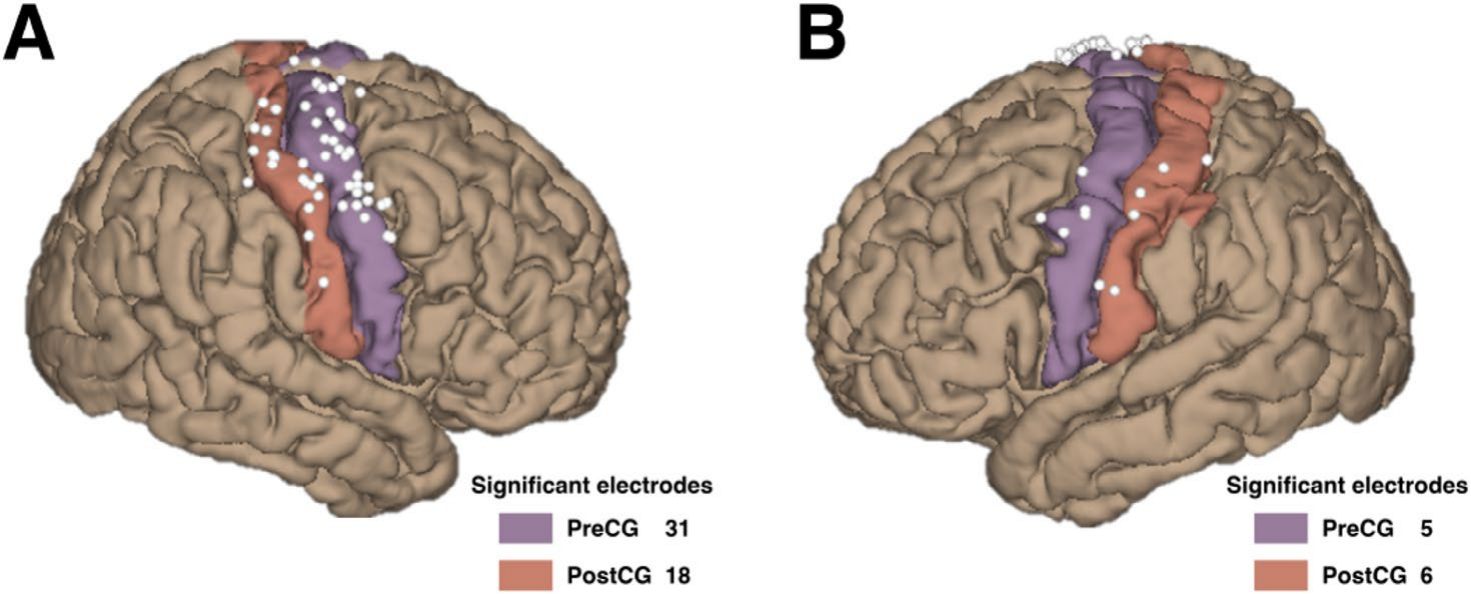
All significant movement‐related electrode locations on the MNI brain. Location of all significant electrodes (white balls) on the MNI152 brain. The selected region of interest (ROI) is defined by colored cortex regions, including the precentral gyrus (PreCG, purple) and postcentral gyrus (PostCG, orange).

In total, 568 electrodes were included among 11 patients, and 139 electrodes were tested inside the ROI (Table 2). Thus, 60 electrodes showed significant HGAs in the grasping task (*p* < 0.05), classified into 36 electrodes in the PreCG and 24 in the PostCG. These electrodes were projected on the MNI brain (Figure 2).

**TABLE 2:** Summary of electrodes in ROI.

Patient	Tested electrodes (*n*)	Significant electrodes (*n*)	Nonsignificant electrodes (*n*)
1	8	6	2
2	19	1	18
3	16	11	5
4	10	9	1
5	14	3	11
6	8	2	6
7	11	2	9
8	18	14	4
9	8	3	5
10	11	2	9
11	16	7	9
Total	139	60	79

### 3.2 Temporal Dynamics of HGA During a Single Movement Trial

**FIGURE 3:**
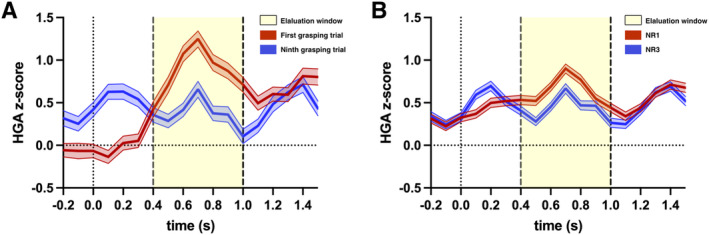
Temporal dynamics of HGA during movement. The mean and ± 2 standard error of the mean (SEM) with a 100‐ms interval for grasping trials (A: the first grasping trial shown in red and the ninth grasping trial in blue) and for merged trials (B: nonresting [[NR]]1 shown in red and NR3 in blue).

Looking at the time course of a single movement in 100‐ms steps showed a substantial HGA increase for the first grasping trial 300 ms after the cue. The HGA of the first trial (Figure 3A, red line) reached a grand average increase of 1.25 z‐score at 700 ms across significant movement locations. The ninth trial (Figure 3A, blue) had a lifted premovement HGA from the previous trial and only a minor peak of 0.66 z‐score at 700 ms. For merged trials for grasping under NR1 and NR3 groups, the HGA standard error overlapped for pre‐ and postmovement periods but demonstrated an apparent attenuation in NR3 for the evaluation period between 400 and 1000 ms (Figure 3B). Again, the HGA peaked at 700 ms, with the HGA in NR1 being higher than NR3, clearly outside the SEM. Table S1 provides detailed information on HGA z‐scores in 100‐ms steps during a single movement trial.

### 3.3 Temporal Dynamics of High Gamma Power by Grasping Repetitions

An HGA z‐score sample was obtained per trial for each significant ECoG location, yielding 600 samples for each “Grasping” trial and “Block.”

**FIGURE 4:**
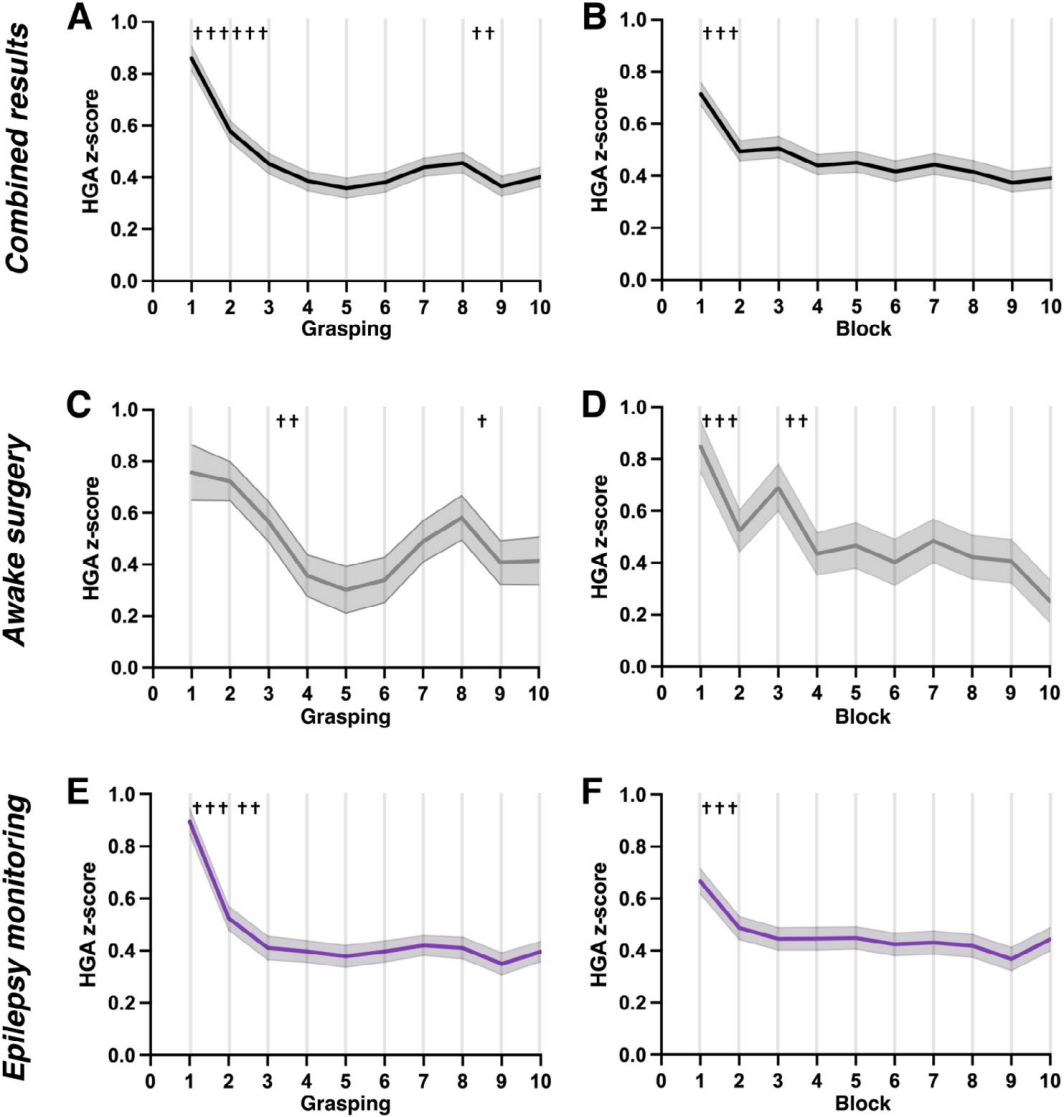
Temporal dynamics of HGA. The mean and ±2 standard error of the mean (SEM) were plotted for consecutive grasping trials and over blocks under three conditions. For consecutive grasping trials (A) and over blocks (B) in combined results from both awake surgeries and epilepsy monitoring based on 1200 trials, crosses indicate significant attenuation (^†††^ *p* < 0.001, ^††^ *p* = 0.008). For consecutive grasping trials (C) and over blocks (D) in awake surgeries based on 320 trials, crosses indicate significant attenuation (C: ^††^ *p* = 0.003, ^†^ *p* = 0.04; D: ^†††^ *p* < 0.001, ^††^ *p* = 0.002). For consecutive grasping trials (C) and over blocks (D) in epilepsy monitoring based on 880 trials, crosses indicate significant attenuation (^†††^ *p* < 0.001, ^††^ *p* = 0.003).

The mean HGA z‐score in each grasping trial was attenuated until the fifth grasping, with a rebound rise afterward and further decay by the ninth grasping (Figure 4A). Comparisons of subsequent trials demonstrated significant attenuation from the first to second trial (Figure 4A; adjusted *p* < 0.001, Dunn's multiple comparison test for nine tests) and from the second to third trial (Figure 4A; adjusted *p* < 0.001, Dunn's multiple comparison test for nine tests), and later from the eighth to ninth trial (Figure 4A; adjusted *p* = 0.008, Dunn's multiple comparison test for nine tests). The mean HGA z‐score in each block was gradually attenuated until the ninth and tenth grasping (Figure 4B). Comparisons of subsequent blocks demonstrated that HGA z‐scores were significantly lower after the first block (Figure 4B; adjusted *p* < 0.001, Dunn's multiple comparison test for nine tests), but no significant differences were observed in the following blocks. A significant difference in the mean HGA z‐score between the eighth and ninth trials was identified in the grasping trials of the awake surgery cohort (Figure 4C), consistent with combined results (Figure 4A). Regarding blocks, significant differences were found between the first and second blocks as well as between the third and fourth blocks, but none were detected afterward (Figure 4D). The trend in the epilepsy monitoring cohort closely matched that of the combined results, except for the comparison between the eighth and ninth grasping trials (Figure 4E,F). HGA z‐scores for each “Grasping” trial and “Block” are detailed in Table S1.

### 3.4 Attenuated HGA Locations in Individual Patients

**FIGURE 5:**
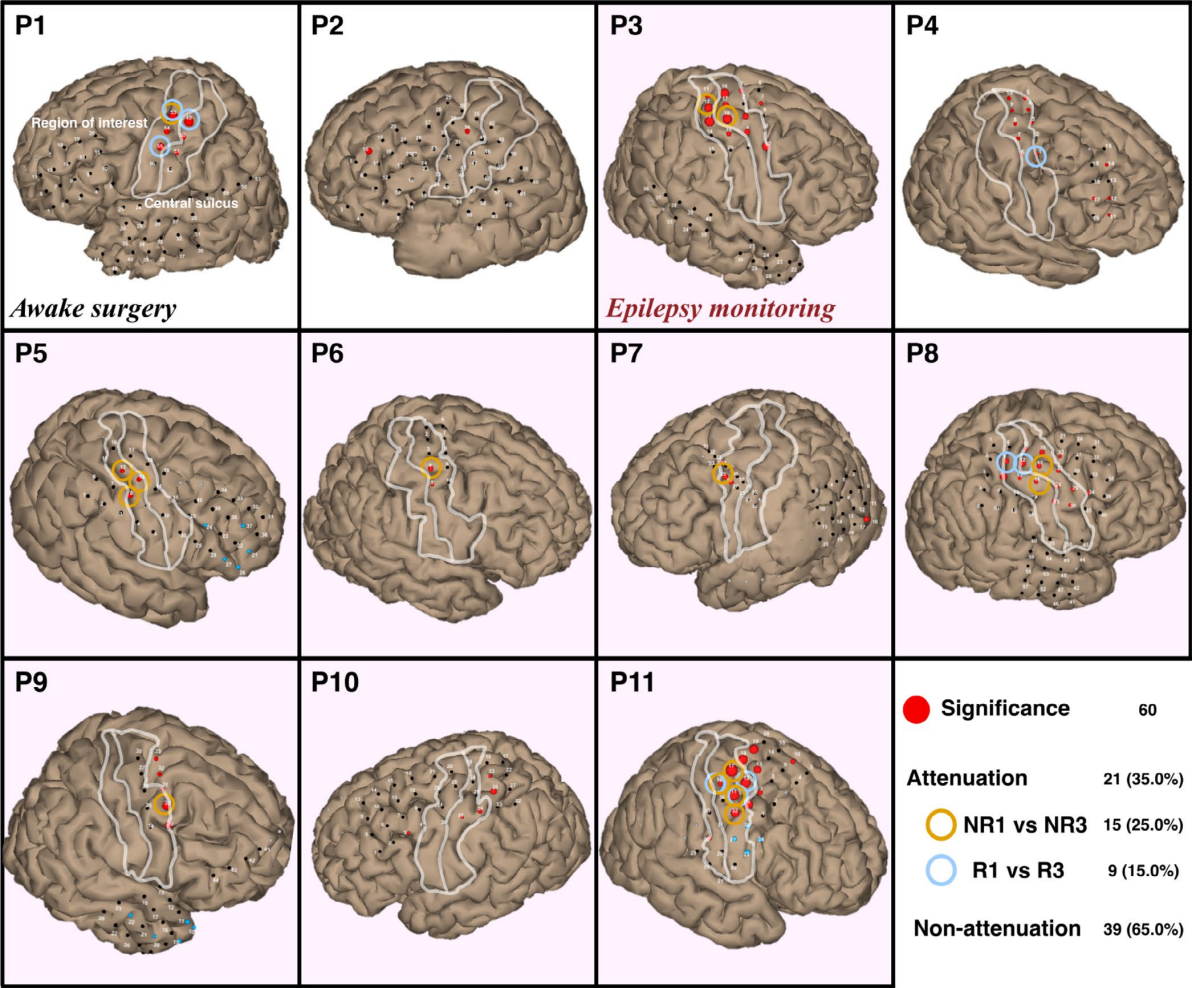
Overview of short‐ and long‐term attenuated locations. The ROI is highlighted with white borders. A channel was defined as short‐term attenuation if nonresting (NR) group NR3 was significantly lower than NR1 (orange circles). A channel was defined as short‐term attenuation if resting (R) group R3 was significantly lower than R1 (light blue circles). Red bubbles indicate significant HGA during movement compared to rest.

Attenuated HGA locations were obtained for each significant movement location in each patient's ROI. Thus, NR1 against NR3, and R1 against R3 were tested, with 90 and 81 samples per group, respectively. Based on a Kruskal–Wallis test with Dunn's multiple comparison test, 25% or 15 out of 60 electrodes in the ROI of 8 patients attenuated significantly from NR1 to NR3, indicating short‐term attenuation (Table 3 and Figure 5). On the other hand, HGA in nine electrodes (15.0%) significantly attenuated from R1 to R3 in 4 patients, indicating long‐term attenuation. Stratifying the cohort referring to awake surgery confirmed the short‐ and long‐term attenuated electrodes with 6.25% and 25.0% of tested locations, respectively, while epilepsy monitoring did 31.82% and 11.36%. The results of patient 1 (a case of awake surgery) and patient 11 (a case of epilepsy monitoring) are described in (Figures S2 and S3), respectively, as representative cases.

**TABLE 3:** Number of short‐term (Grasping) and long‐term (Block) attenuated electrodes with high gamma activity.

Mapping	Patient	Significant electrodes (*n*)			
		Grasping electrodes (*n*)		Block electrodes (*n*)	
		Attenuation NR1 vs NR3	Nonattenuation	Attenuation R1 vs R3	Nonattenuation
Awake surgery	1	1	5	3	3
	2	0	1	0	1
	4	0	9	1	8
	Total	1	15	4	12
Epilepsy monitoring	3	2	9	0	11
	5	3	0	0	3
	6	1	1	0	2
	7	1	1	0	2
	8	2	12	2	12
	9	1	2	0	3
	10	0	2	0	2
	11	4	3	3	4
	Total	14	30	5	39
All		15	45	9	51

### 3.5 MNI Locations of Attenuated HGA

**FIGURE 6:**
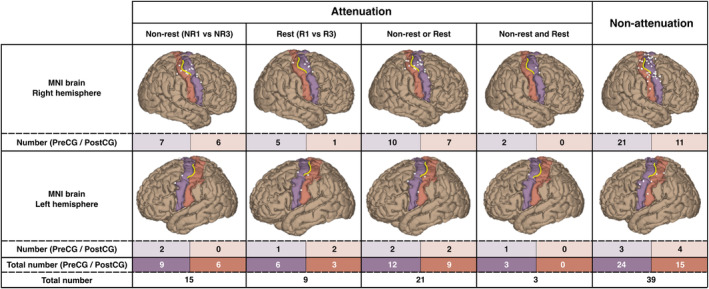
Spatial characteristics of MNI brain of attenuated and nonattenuated high gamma power. Location of all significant electrodes (white balls) on the MNI152 brain stratifying into each hemisphere, attenuation, nonattenuation. The selected region of interest (ROI) is defined by colored cortex regions, including the precentral gyrus (PreCG, purple) and postcentral gyrus (PostCG, orange). The hand‐knob region in the central sulcus was outlined with a yellow line.

The MNI location of each electrode was used to merge all attenuated HGA locations onto a single brain (Figure 6). Overall, the total number of attenuated electrodes in the PreCG was larger than in the PostCG for both NR or R groups, although Fisher's exact test revealed no significant difference in either group (NR: *p* > 0.99; R: *p* = 0.73). While nonattenuated electrodes were distributed across a relatively wide area of the PreCG and PostCG, attenuated electrodes appeared visually concentrated around the hand‐knob region of the PreCG and the adjacent region of the PostCG gyrus. Nevertheless, there was no significant difference in the distance of electrodes from the deepest portion of the hand‐knob “sigma” between attenuated and nonattenuated electrodes in the NR groups (median value = 20.47 mm vs. 23.44 mm, *p* = 0.58) or in the R groups (median value = 17.81 mm vs. 23.44 mm, *p* = 0.32).

## 4 Discussion

The present study demonstrated attenuated HGA z‐scores of significantly activated sensorimotor locations over time for individual grasping trials and over repeated blocks (Figure 4). These findings support the hypothesis that HGA weakens over time by repetitive grasping movements in the sensorimotor area, which is in line with previously reported ECoG HGA attenuation in other functional areas (Matsuzaki et al. 2012; Rangarajan et al. 2020) as well as the amplitude of motor‐related cortical potentials in scalp EEG (Dirnberger, Duregger, Trettler, et al. 2004; Dirnberger, Duregger, Lindinger, et al. 2004). Moreover, the HGA attenuation plateaued and was sustained late. This trend is explained by a previous fMRI study, which showed a nonlinear model of the motor cortex's high‐frequency band amplitude through repetitive hand movements (Hermes, Siero, et al. 2012). Since broadband activity is associated with neuronal population firing rate (Hermes, Siero, et al. 2012; Miller et al. 2009; Ray and Maunsell 2011) and strongly correlates with the fMRI signal (Hermes, Miller, et al. 2012; Lachaux et al. 2007; Niessing et al. 2005), the current result implies that the neural activity is decreased for repeated grasping as well.

Furthermore, comparing two consecutive grasping trials demonstrated that HGA z‐scores significantly reduced from the first to second and second to third, but not later (Figure 4). The lower the HGA z‐score is, the weaker the contribution to the effect size of the statistical test. Therefore, the goal was to collect sufficient data for a reliable statistical analysis with high statistical power, but on the other hand, to minimize the mapping time to reduce the time for the operator the burden for the patient and to achieve a high effect size (i.e., *R* ^2^ value), showing a clear mapping output for interpretation. Thus, any attenuated HGA period should be omitted if not required by the statistical analysis. Given the analysis constraints, the number of grasping per block and total blocks should be designed so that a minimum of attenuated locations occurs until the end of the mapping. Taking together the attenuated electrodes of all patients, short‐term attenuation appears mainly for the first three grasping trials within a block and then reaches a plateau. In contrast, the long‐term attenuation strongly appears after the first block and then slightly continues. Hence, having more blocks with fewer grasping trials is preferable to achieve the most substantial effect size, which can be achieved with three grasping trials over ten blocks. When tasks incorporate multiple alternative movements within each block, interleaving different movement types introduces resting periods between the hand movement trials (Jensen et al. 2023, 2024). This approach could midgate attenuation in NR groups, thereby minimizing its impact on HGA mapping. Nevertheless, our findings support the strength of HGA mapping to rapidly identify significant task‐related locations in a shorter time than ECS mapping (Kapeller et al. 2015; Ogawa et al. 2014, 2017). Furthermore, stereoelectroencephalography (SEEG) has become a more widely used clinical tool in epilepsy monitoring unit than ECoG. A recent study demonstrated that task‐based electrophysiological mapping using broadband changes in the SEEG signals reliably localized activity to sensorimotor cortical regions (Jensen et al. 2024). Our findings regarding HGA attenuation could potentially be applied to HGA mapping using SEEG.

From 60 identified grasping locations, 15 (25%) and 9 (15%) demonstrated attenuated HGA over the short‐term NR groups and long‐term R groups, respectively (Figure 5). It is notable that when dividing the patients in awake surgery (*N* = 3) and epilepsy monitoring (*N* = 8) subgroups, the attenuated short‐ and long‐term locations are 6.25% and 25.0%, and 31.82% and 11.36%, respectively. The predominant tendency of short‐term attenuation within NR groups mainly occurred in epilepsy patients, with 14 of 44 (32%) locations. This short‐term attenuation rate fits the previous ECoG study of attenuation for a working memory, which reported 17 of 50 electrodes (34%) (Merzagora et al. 2014). In contrast, the awake surgery cases showed a higher rate of long‐term attenuation over R groups, with 4 of 16 (25%) locations in Table 3. Despite the small sample size, the awake surgery cases indicate an opposite trend for short‐ and long‐term attenuation. Additionally, two of three awake surgery cases exhibited baseline attenuation (Figure S4). These differences might be attributed to the side effects of the anesthetic drugs, generally lowering HGA and fewer significant HGA locations during short‐term rest paradigms (Nourmohammadi et al. 2023). These findings suggest that the mapping protocol may need to be adjusted for the operating room or, as a tradeoff, rebalanced to five blocks of six grasping trials each, considering both use cases.

Surprisingly, grasping showed a significant augmentation of HGA between the eighth and ninth grasping. This phenomenon is less frequently reported than repetition suppression but is known as repetition enhancement by previous studies using single‐unit recordings (Baylis and Rolls 1987; Miller, Li, and Desimone 1993) and fMRI (Henson, Shallice, and Dolan 2000; Müller et al. 2013). Repetition enhancement responds later while repetition suppression did earlier, possibly due to their different functional roles (Barron, Garvert, and Behrens 2016; de Gardelle et al. 2013). Although the underlying mechanism of repetition enhancement is poorly understood, the anesthetic drugs might affect the occurrence of repetition enhancement, considering that there is no repetition enhancement in the epilepsy monitoring subgroup.

Although significant HGA attenuated locations occurred more frequently in the PreCG compared with the PostCG, the differences in the number of electrodes were not statistically significant in either the NR or R groups. Furthermore, the spatial distribution of attenuated electrodes across patients on the MNI brain highlighted their predominant localization around the hand‐knob region of the PreCG and the adjacent region of the PostCG (Figure 6), consistent with findings from the fMRI studies demonstrating attenuation associated with repetitive hand motor tasks (Hermes, Siero, et al. 2012; Valyear et al. 2012). However, quantitative analysis of the electrode distance from the deepest portion of the hand‐knob “sigma” did not reveal the statistical significance between attenuated and nonattenuated electrodes in either the NR or R groups. These findings may imply the need for careful consideration of attenuation effects during repetitive grasping tasks, regardless of the area surrounding the hand knob.

The attenuation effect in the HGA in the sensorimotor cortex is most prominent between 0.4 and 1.0 s after the grasping instruction. The HGA of the first grasping peaked at the same time as the ninth trial (Figure 3A) but with a baseline effect at the beginning. This baseline effect occurred because the first grasping was the initial movement following a prolonged rest period of 12 s, resulting in a flat HGA at the start. In contrast, ninth grasping included residual HGA of preceding hand‐opening movement associated with previous trials, leading to a difference at 0.2 s, which is outside the evaluation window (0.4–1.0 s) (Figure 3A,B). However, our study specifically focuses on HGAs during hand‐closing movements. Therefore, we excluded HGAs outside the evaluation window and found that the temporal dynamics support the appropriateness of our evaluation window for measuring HGA. Notably, the HGA peak of the first trial is higher and rises earlier compared to later trials, confirming the findings from previous studies regarding working memory and face recognition (Merzagora et al. 2014; Rangarajan et al. 2020).

Several limitations of the present study must be addressed. First, the number of patients was limited to 11. The small sample size may limit generalizability, and a large cohort is needed to validate our findings. Nevertheless, this study is the first to demonstrate the annotation effect and temporal dynamics using HGA z‐scores in the sensorimotor area, providing a basis for inferences. Second, our results combined the data of HGA mapping using different methods, such as awake surgery and epilepsy monitoring. The data acquisition is slightly different in the operation room for awake surgery and at the patient's bedside for monitoring. The method used for the co‐registration of images was also different. However, we applied the previously published co‐resignation method to awake surgery (Dalal et al. 2008; Gupta et al. 2014), and the results showed significant and attenuated electrodes without apparent discrepancy to those of epilepsy monitoring. Third, we used FLAIR images for anatomical MRI images to co‐registered with postoperative CT images in patient 5. FLAIR images still enabled us to visualize the brain surface's anatomy and successfully show the positional relationship between electrodes and the anatomy of the sensorimotor cortex (Billot, Magdamo, et al. 2023; Billot, Greve, et al. 2023; Gopinath et al. 2023; Iglesias et al. 2023). Fourth, the left hemisphere's limited electrode count may influence spatial characteristics. However, combining data from both hemispheres showed a general pattern in these spatial characteristics (Figure 6). At the same time, only the PreCG and PostCG were included in ROI for sensorimotor mappping; other relevant cortical areas—such as the supplementary motor area (Fuelscher et al. 2019), inferior frontal gyrus (Kilner et al. 2009; Press, Weiskopf, and Kilner 2012), and inferior parietal lobe (Chong et al. 2008)—could be explored in the future studies. Fifth, the current study did not confirm sensorimotor functions using ECS. Future studies incorporating ECS validation could strengthen our findings.

## 5 Conclusion

The present study showed that repetitive grasping tasks attenuated the HGA of significant electrodes in the sensorimotor area over time. Furthermore, the short‐term repetition of grasping within a block tended to be more affected than the number of blocks. Notably, this is mainly the case in the epilepsy monitoring unit when patients are fully awake. Hence, the optimal mapping paradigm may slightly differ for awake surgeries. Considering these findings can further improve the usability of ECoG mapping in terms of clearer results in the most reasonable mapping time.

## Conflicts of Interest

C.K. and M.J. are employees of g.tec Medical Engineering GmbH. C.G. is the CEO of g.tec Medical Engineering GmbH. The remaining authors declare that the research was conducted in the absence of any commercial or financial relationships that could be construed as a potential conflicts of interest.

## Supporting information


**Figure S1.** Flow of co‐registration in cases of awake surgery and epilepsy monitoring. The postoperative CT scans were co‐registered to the anatomical MRI scans using the cortiQ Montage Creator software. Then, electrodes in the CT were projected to the cortex surface.

**Figure S2.** The result of *P1* (*awake surgery*) with short‐term and long‐term attenuated locations. The ROI is highlighted with white borders. A channel was defined as short‐term attenuation if nonresting (NR) group NR3 was significantly lower than NR1 (orange circles). A channel was defined as short‐term attenuation if resting (R) group R3 was significantly lower than R1 (light blue circles). Red bubbles indicate significant HGA during movement compared to rest.

**Figure S3.** The result of *P11* (*epilepsy monitoring*) with short‐term and long‐term attenuated locations. The ROI is highlighted with white borders. A channel was defined as short‐term attenuation if nonresting (NR) group NR3 was significantly lower than R1 (orange circles). A channel was defined as short‐term attenuation if resting (R) group R3 was significantly lower than R1 (light blue circles). Red bubbles indicate significant HGA during movement compared to rest.

**Figure S4.** The distribution and statistical difference of baseline HGA. Violin plots illustrating the HGAs of the baselines before each block for P1 to P11 (A). Statistical comparison of median HGAs between block 1–3 and block 7–9 using the Mann–Whitney *U* test was shown (B). Crosses indicate significant attenuation (+++: *p* < 0.001, ++: *p* < 0.01, +: *p* < 0.05).

**Table S1.** Detailed information on HGA z‐scores for each “Grasping” trial and “Block,” as well as HGA z‐scores in 100 ms intervals during a single movement trial.

## Data Availability

Anonymized data of calculated HGA presented in this article are provided in Table S1. The authors will review requestable requests for additional data to determine whether they can be fulfilled following the privacy restrictions of each participating institution. Requests for additional materials related to this work should be directed to T.S.
