# Supplementary figures and images for: Attenuation of High Gamma Activity by Repetitive Motor Tasks
Source: Hum Brain Mapp. 2025 Feb 7;46(2):e70153. doi: 10.1002/hbm.70153 (PMC11803456; doi:10.1002/hbm.70153)

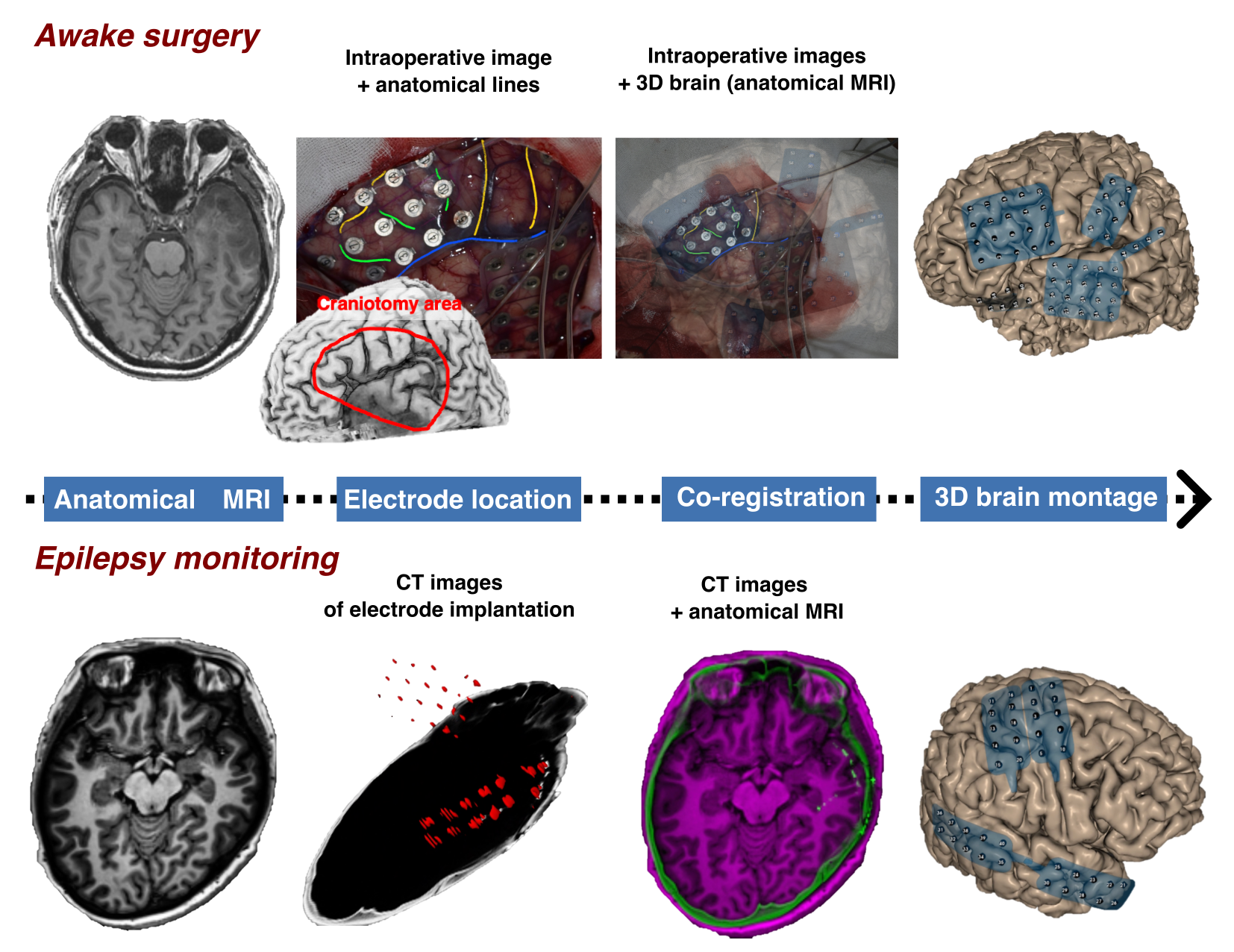

Supplement: Supplementary file 1 — Figure S1. Flow of co‐registration in cases of awake surgery and epilepsy monitoring. The postoperative CT scans were co‐registered to the anatomical MRI scans using the cortiQ Montage Creator software. Then, electrodes in the CT were projected to the cortex surface. [file HBM-46-e70153-s003.tiff]

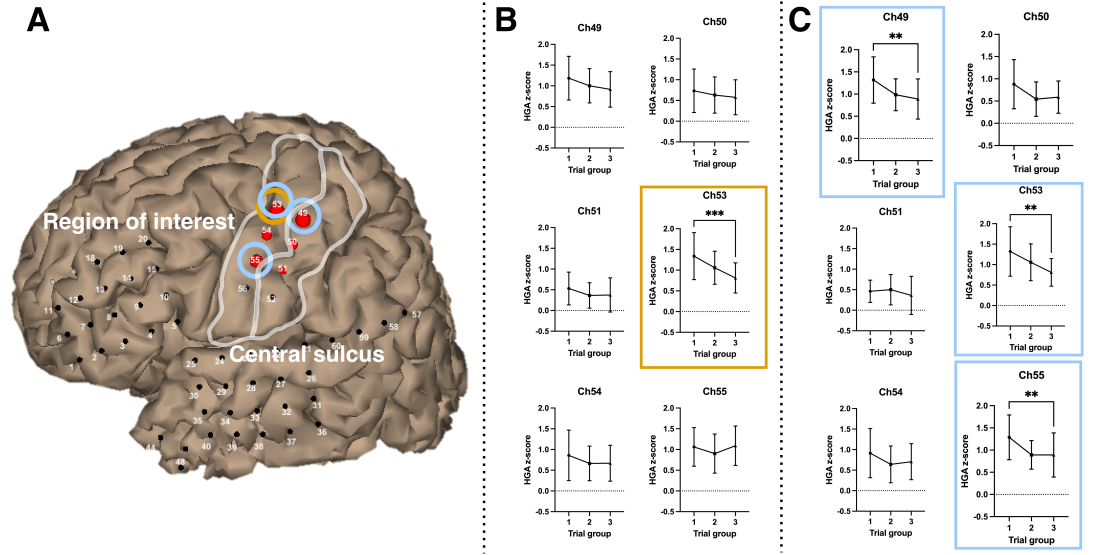

Supplement: Supplementary file 2 — Figure S2. The result of P1 (awake surgery) with short‐term and long‐term attenuated locations. The ROI is highlighted with white borders. A channel was defined as short‐term attenuation if nonresting (NR) group NR3 was significantly lower than NR1 (orange circles). A channel was defined as short‐term attenuation if resting (R) group R3 was significantly lower than R1 (light blue circles). Red bubbles indicate significant HGA during movement compared to rest. [file HBM-46-e70153-s005.tiff]

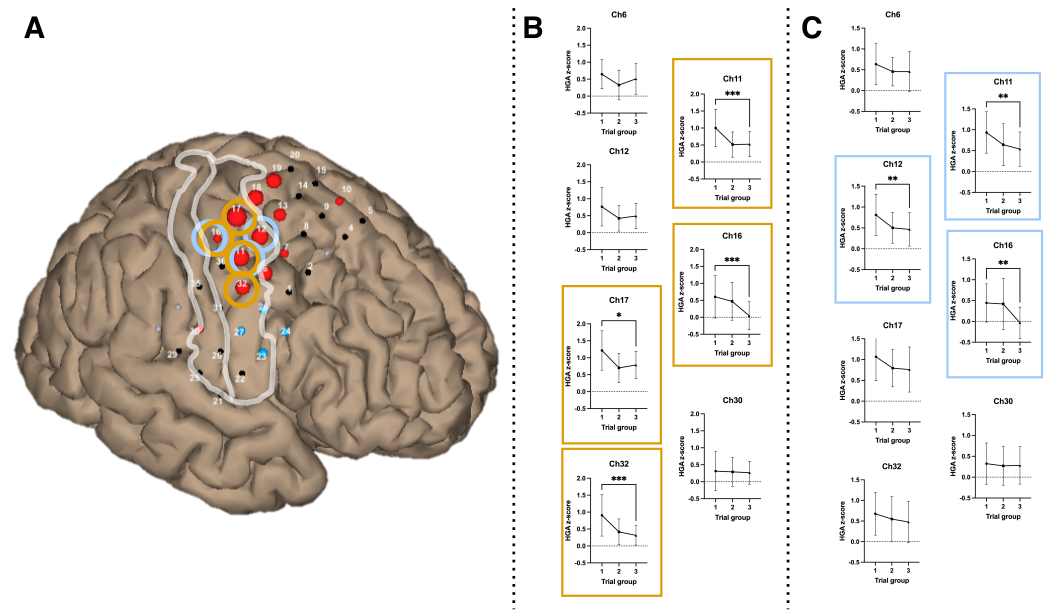

Supplement: Supplementary file 3 — Figure S3. The result of P11 (epilepsy monitoring) with short‐term and long‐term attenuated locations. The ROI is highlighted with white borders. A channel was defined as short‐term attenuation if nonresting (NR) group NR3 was significantly lower than R1 (orange circles). A channel was defined as short‐term attenuation if resting (R) group R3 was significantly lower than R1 (light blue circles). Red bubbles indicate significant HGA during movement compared to rest. [file HBM-46-e70153-s004.tiff]

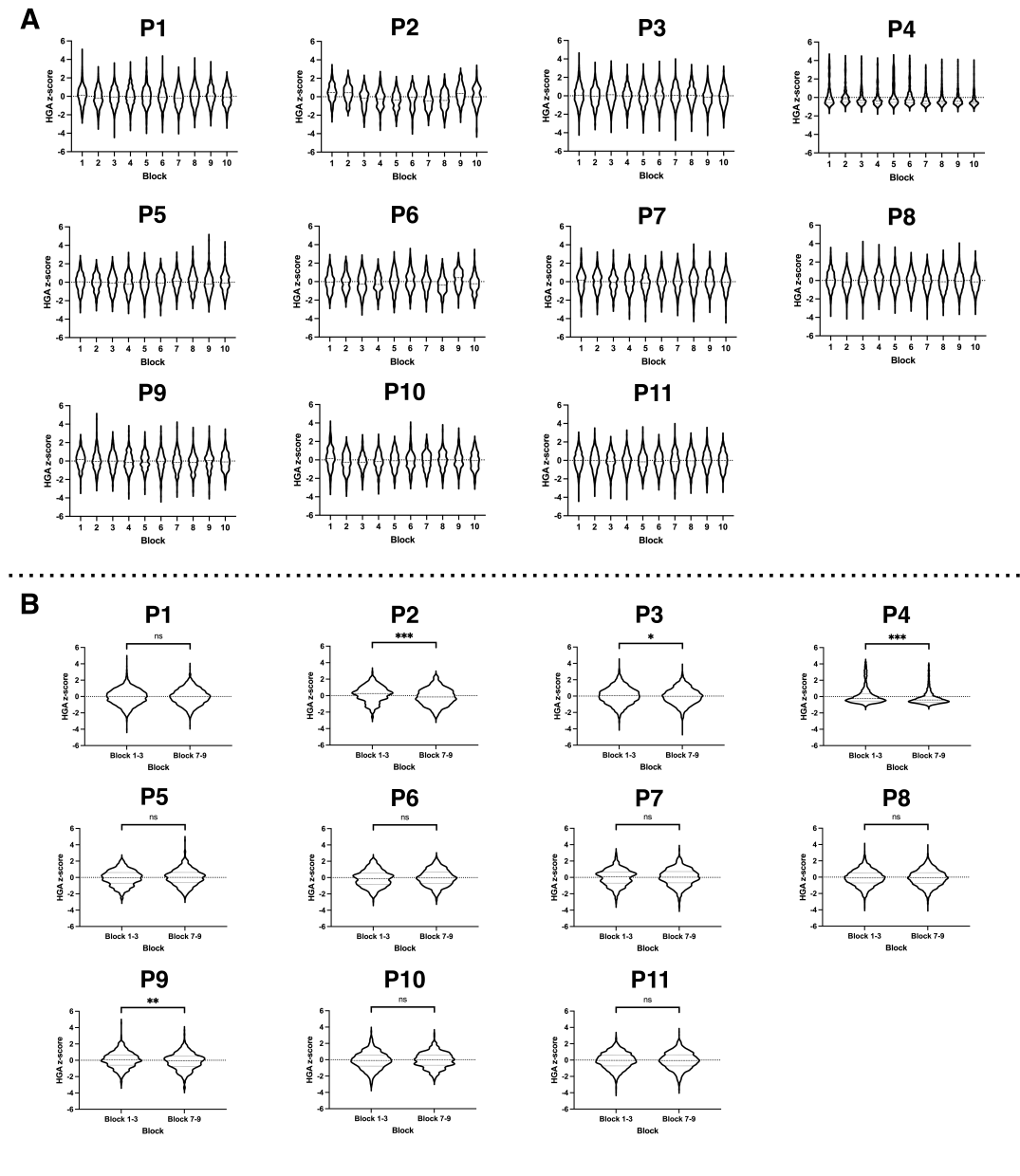

Supplement: Supplementary file 4 — Figure S4. The distribution and statistical difference of baseline HGA. Violin plots illustrating the HGAs of the baselines before each block for P1 to P11 (A). Statistical comparison of median HGAs between block 1–3 and block 7–9 using the Mann–Whitney U test was shown (B). Crosses indicate significant attenuation (+++: p < 0.001, ++: p < 0.01, +: p < 0.05). [file HBM-46-e70153-s002.tiff]
